# Electricity use is associated with residents’ vital data and lifestyles: observational study using an IT health support system in Japan

**DOI:** 10.1038/s41598-020-74359-4

**Published:** 2020-10-13

**Authors:** Keiji Yasukawa, Yukio Ishihara, Fumi Hirayama, Megumi Nakanishi, Hideo Utsumi, Susumu Koyama

**Affiliations:** 1 Innovation Center for Medical Redox Navigation, Kyushu University, 3-1-1 Maidashi, Higashi-ku, Fukuoka, 812-8582 Japan; 2 Laboratory of Advanced Pharmacology, Faculty of Pharmaceutical Sciences, Daiichi University of Pharmacy, 22-1 Tamagawa-machi, Minami-ku, Fukuoka, 815-8511 Japan; 3 Present Address: General Information Processing Center, Shimane University, 1060 Nishikawatsu-cho, Matsue-shi, Shimane, 690-8504 Japan; 4 Present Address: School of Pharmaceutical Sciences, University of Shizuoka, 52-1 Yada, Suruga-ku, Shizuoka, 422-8526 Japan

**Keywords:** Information technology, Health care, Lifestyle modification

## Abstract

Motivated by developments in information technology, recording personal parameters with health devices is effective in health promotion. Today’s indoor individual lifestyles often involve using electrical appliances. We developed a health support system combined with wireless electricity monitoring and investigated whether electricity use is associated with residents’ vital data and lifestyles. We recruited 116 participants in February 2013. Their vital and electricity use data were collected daily. They completed a self-administered questionnaire. Among participants living alone, electricity from 20 February to 11 March 2013 was negatively associated with high-density lipoprotein (HDL) (*P* = 0.008) and positively associated with low-density lipoprotein (LDL) (*P* = 0.007) and neutral fat (*P* = 0.020) levels. Among all participants, electricity use was negatively associated with vegetable intake (*P* = 0.044) and step count (*P* = 0.040). Temperature sensitivity in winter was negatively associated with the LDL/HDL ratio for both men and women. For men, temperature sensitivity in winter was negatively related with alcohol intake; for women, it was positively related to body fat percentage and abdominal circumference and negatively correlated to vegetable intake. Temperature sensitivity in summer was positively associated with vegetable intake for men and women. In conclusion, electricity use was related to vital data and lifestyles and influenced by temperature.

## Introduction

Chronic diseases, also known as non-communicable diseases (such as diabetes, stroke, hypertension, arteriosclerosis, and chronic respiratory diseases), were responsible for 38 million (68%) of the world’s 56 million deaths in 2012^1^. Inappropriate dietary patterns, insufficient physical activity, and harmful use of alcohol and tobacco use are believed to be major risk factors for these diseases^1,2^. Hyperlipidemic conditions, such as low levels of high-density lipoprotein (HDL) cholesterol, are reported to be closely associated with the incidence of cardiovascular disease^3,4^. Preventing lifestyle-related diseases requires improving lifestyles and continuous efforts to maintain them. Recording the parameters on an individual basis using such health devices as sphygmomanometers and pedometers, which is motivated by the development of information technology (IT), is effective in promoting health^5–7^.

In addition to the development of IT, the desire to conserve electricity through environmental concerns has promoted the development of home electricity monitors (in-home displays), which provide visual information about household electricity usage^8,9^. Indoor activities frequently involve various kinds of electrical appliances; thus, wireless monitoring of domestic electricity use is effective in characterizing people’s behaviour at home^10^. The time when elderly people begin watching television appears to be useful in diagnosing poor health^11^. Home sensing technology, including electricity use, has been applied to assess health outcomes and recovery following hip and knee replacement in the United Kingdom^12^. Thus, we considered that the amount of domestic electricity use would be related to residents’ vital data and lifestyles. Accordingly, we developed a home health support system using wireless electricity monitoring; we undertook an observational study to test our hypothesis about the relationship between domestic electricity use and residents’ health. We investigated whether domestic electricity use was linked to residents’ vital data (mean blood pressure, body mass index, waist circumference, and blood lipid levels) and lifestyles (steps taken, total metabolic equivalent tasks [METs], vegetable or fruit intake, number of cigarettes smoked, and amount of alcohol intake) in winter. Domestic electricity use is greatly influenced by outside temperatures; thus, we continued to monitor the residents’ electricity use, vital data, and lifestyles with the home health support system for 1 year; we investigated the effect of outside temperatures on the relationship between domestic electricity use and residents’ vital data and lifestyles.

## Methods

### Ethics statement

Approval of the study protocol was obtained from the Kyushu University Ethical Committee (24-169). After a complete description of the study, all participants provided their written informed consent to take part. All methods were performed in accordance with the relevant guidelines and regulations.

### Participants

We recruited 116 participants living in Kyushu, Japan through an advertisement on the Internet. The inclusion criteria were as follows: (1) healthy adult males and females aged 20–59 years who provided their informed consent; (2) having an Internet environment at home. The exclusion criteria were as follows: individuals who had difficulty in measuring their parameters with health devices and handling the tablet daily owing to physical disease. We excluded 11 people from the data analysis for the following reasons: data from the home health support system in five cases could not be collected owing to difficulty in installing the system; the electricity data in six cases could not obtained owing to problems with the power distribution board.

### Overview of home health support system

**Figure 1:**
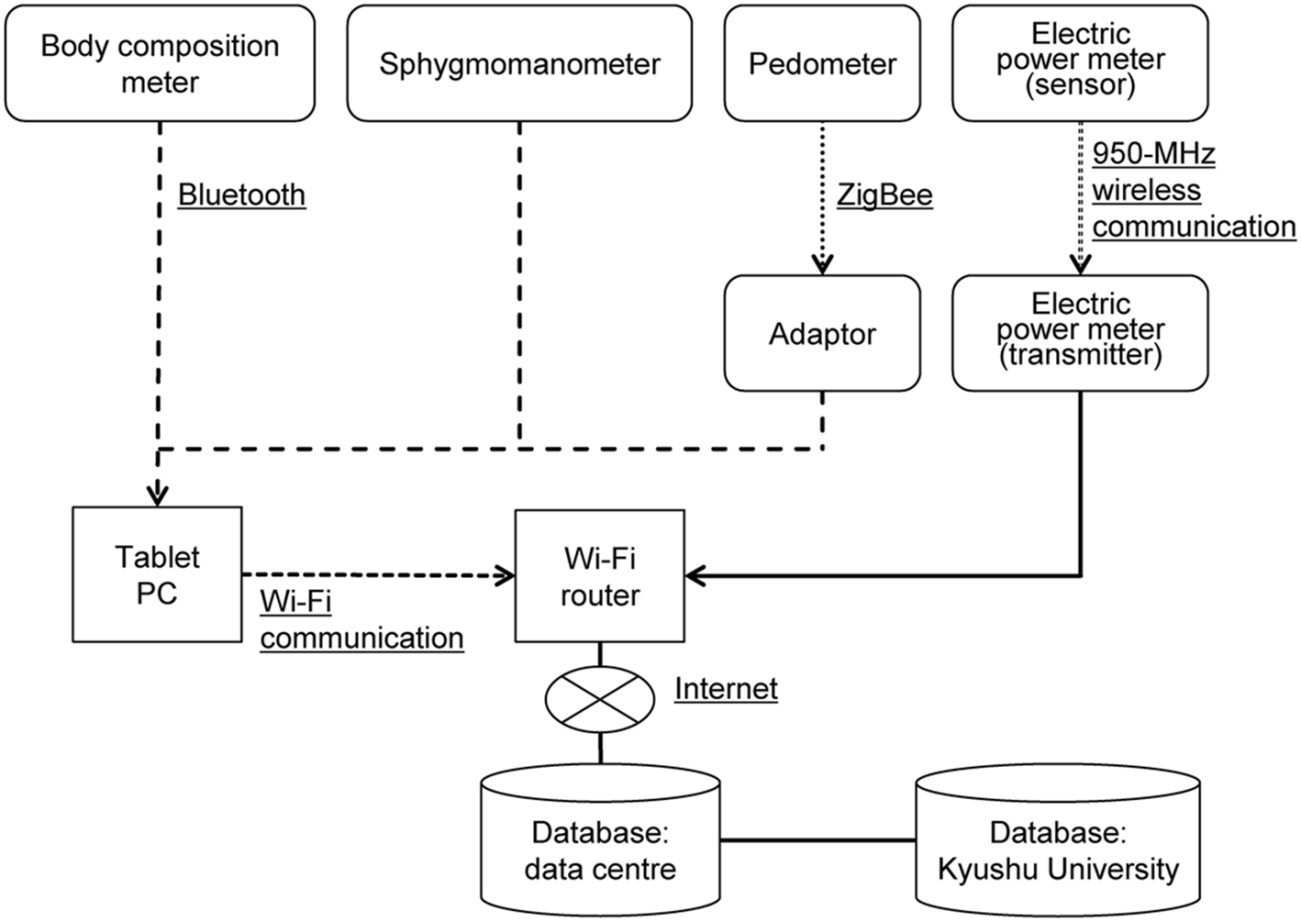
Overview of home health support system.

The devices for the home health support system together with an instruction manual were delivered to the homes of each participant in the first or second week of February 2013. An overview of the home health support system, developed by Kyuden Business Solutions, appears in Fig. 1. A body composition meter (A&D Company, Limited, Tokyo), a sphygmomanometer (A&D Company, Limited, Tokyo), and an adaptor for a pedometer (Omron Corporation, Kyoto) were wirelessly connected to a tablet computer (Toshiba Corporation, Tokyo) using a Health Device Profile protocol for Bluetooth. Data from the body composition meter or sphygmomanometer were automatically transmitted to the tablet computer. The pedometer data were transmitted to the tablet computer via an adaptor. Data stored on the tablet computer, which was wirelessly connected with a LAN router, were transmitted to a data centre (Kyuden Infocom Company, Inc., Fukuoka) over the Internet; that occurred when participants clicked the “Receive” button at the top of the dedicated application, created by Kyuden Business Solutions, installed in the tablet computer. The electric power meter comprised two units: one detected power consumption at 1-min intervals; using ZigBee, it transmitted the data to the other unit, which calculated the average consumption every 10 min and transmitted the information to the data centre via a wireless LAN router and the Internet.

After each participant completed the installation, data sampling from the body composition meter, sphygmomanometer, pedometer, and electric power meter was initiated to confirm that all data from the health devices and electric power meter were properly accumulated by the data server and could be monitored using the tablet via the Internet. Participants were asked to measure their body weight using the body composition meter when they awoke and their blood pressure using the sphygmomanometer when they awoke and before they slept. Participants were asked to have the pedometer with them during waking hours.

Data stored by the server at the data centre did not include information that could identify the individuals. The data were shared with the Kyushu University server, and that computer had no external connection with the Daiichi University of Pharmacy for statistical analysis. Identification numbers were assigned to each participant, and the password-locked electric data of the correspondence table was stored in a computer with no external connection. After checking the system operation for all participants, we used data from 20 February 2013 for the statistical analysis.

### Analysis of domestic electricity use

**Figure 2:**
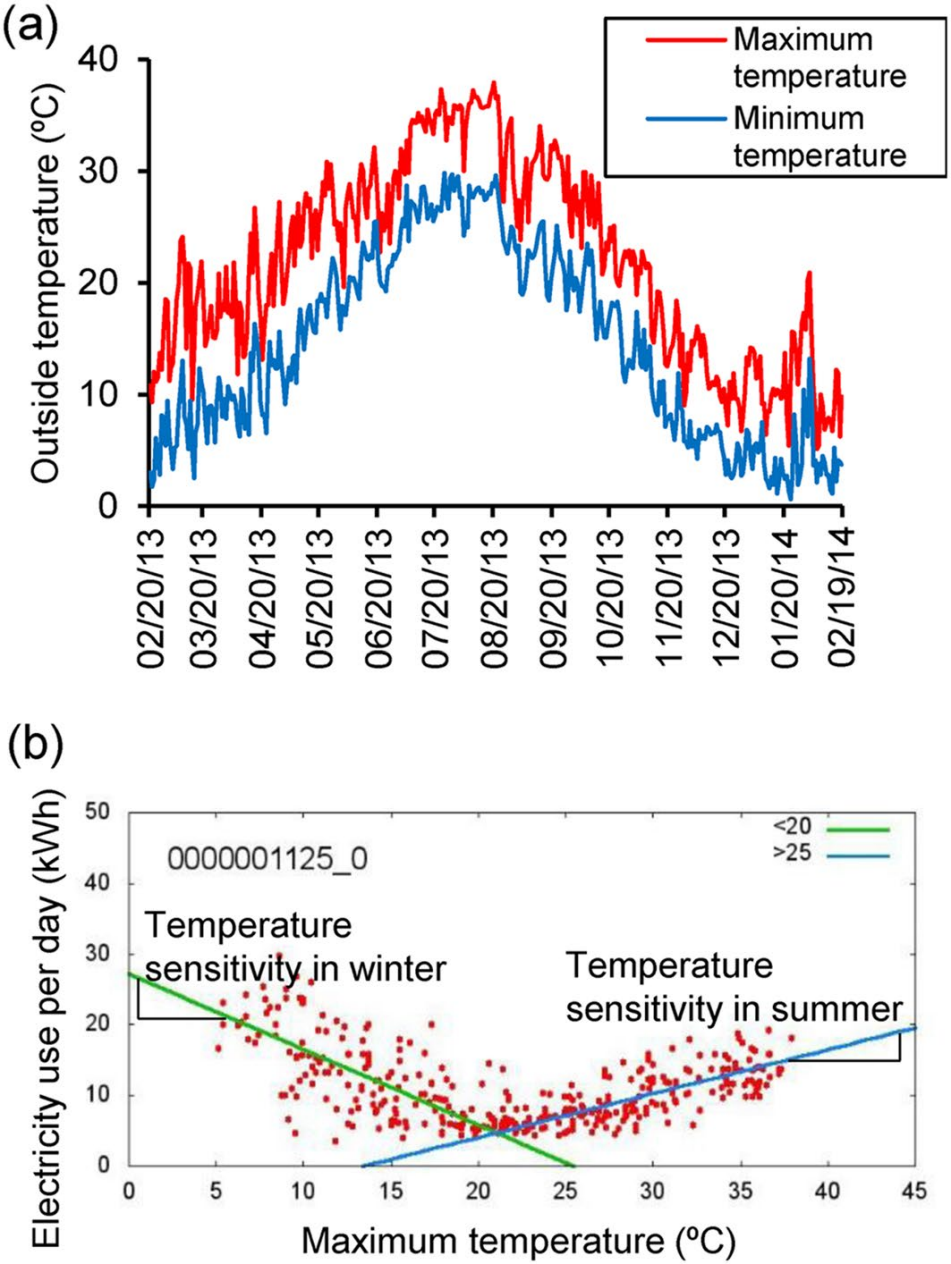
Profile of outside temperature during the data collection period and temperature sensitivity of electricity use. (**a**) Profile of maximum and minimum temperatures during the data collection period in Fukuoka; (**b**) example of temperature sensitivity in winter and summer. In (**a**), the red and blue lines represent maximum and minimum temperatures in Fukuoka from 20 February 2013 to 19 February 2014, respectively. In (**b**), electricity use per day (kWh) is plotted against the corresponding maximum temperature (°C) indicated by red dots. The green and blue lines show the temperature sensitivity in winter and summer, respectively.

We performed two types of analysis of domestic electricity use: one was an analysis of mean electricity use over a short term (20 days); the other was temperature sensitivity of domestic electricity use in winter and summer. With the first type, we used data stored in the server from 20 February to 11 March 2013 (20 days). With the second type, we employed data stored in the server from 20 February 2013 to 19 February 2014. Electricity use in winter increases with decreased temperature, and it rises in summer with increased temperature in most areas of Japan and other countries with hot and cold seasons^13,14^. The maximum and minimum temperatures from 20 February 2013 to 19 February 2014 in Fukuoka, where most of the participants lived, appears in Fig. 2a. We obtained the temperature data from the weather database of the Japan Meteorological Agency (https://www.data.jma.go.jp/gmd/risk/obsdl/index.php). Daily home electricity use was plotted as a function of daily maximum temperature from 20 February 2013 to 19 February 2014; the linear increase in daily domestic electricity use over 25 °C and linear decrease in such use below 20 °C appeared as the daily maximum temperature increases (Fig. 2b). Thus, we defined the positive slope in that figure obtained from the linear fitting over 25 °C as the temperature sensitivity of summer; we defined the negative slope obtained from the linear fitting below 20 °C as the temperature sensitivity of winter.

### Questionnaire

Participants were asked to complete a written questionnaire at the start of the study and every 4 months during the measurement. We restricted the number of participants responding to the questionnaire to one person per household. The structured questionnaire requested information about demographic and lifestyle characteristics, including the following: age; gender; number and age of household members; height; education level; numbers of cigarettes smoked per day; amount of alcohol consumed per month; vegetable and fruit intake per week; inactive periods on weekdays and at weekends; frequency and duration of physical activity; waist circumference; blood lipid (HDL, LDL, and neutral fat) levels (from recent data from regular health examinations or blood tests conducted by the participant’s doctor); recent history of such health conditions as hypertension and diabetes mellitus; and use of home appliances.

### Data for alcohol consumption and vegetable and fruit intake

Information about alcohol consumption and vegetable and fruit intake was obtained using the STEPwise approach to surveillance questionnaire developed by the WHO^15^. Participants who drank alcohol (sake, *shochu* [Japanese spirit], beer, whisky, or wine) at least once the previous month were asked to report the average quantity consumed for each beverage. For each beverage, the alcohol intake (g/day) was calculated by multiplying the average quantity of beverage taken (ml) per day and ethanol concentration (%) with the specific gravity of ethanol (0.8). The following conventional measurements of quantity were adopted: sake (180 ml/cup), *shochu* (90 ml/cup), beer (500 ml/bottle), whisky (60 ml/glass), and wine (120 ml/glass). The total alcohol intake (g/day) was the sum of the amounts for the five beverages. Finally, the monthly alcohol consumption for an individual was the product of the total alcohol intake (g/day) multiplied by their frequency of drinking (days per month).

Participants were asked, “In a typical week, how many days do you eat fruit?” and “How many servings of fruit do you eat on one of those days?” One serving was equivalent to one medium-sized piece of apple, banana, or orange. One serving was a 80 g of chopped, cooked, canned fruit; 100 ml of fruit juice was considered a comparable capacity for one serving. Similarly, participants were asked about vegetable consumption: “In a typical week, how many days do you eat vegetables?” and “How many servings of vegetables do you eat on one of those days?” One serving was equivalent to 80 g of raw green leafy vegetables (such as spinach and lettuce). One serving was 80 g of other cooked, chopped, or raw vegetables; 100 ml of vegetable juice was considered a comparable capacity for one serving.

### Measuring physical activity

Physical activity was measured using the International Physical Activity Questionnaire (IPAQ)^16^, which was developed as a tool for cross-national monitoring of physical activity. There are short and long versions of the questionnaire. The short version comprises nine items identifying the time spent over the last 7 days with walking (frequency, duration, and pace), vigorous activity (frequency and duration), moderate activity (frequency and duration), and sedentary activity (duration on weekdays and at weekends). The IPAQ incorporates a scoring mechanism, whereby each activity is assigned an intensity code expressed in terms of METs. An MET is the ratio of the metabolic rate during an activity compared with the metabolic rate at rest. For each type of activity, we calculated the weighted MET-minutes per week as follows:

- Walking MET-minutes/week = 3.3 × walking minutes × walking days.
- Moderate MET-minutes/week = 4.0 × moderate intensity activity minutes × moderate days.
- Vigorous MET-minutes/week = 8.0 × vigorous intensity activity minutes × vigorous intensity days.

We computed the total physical activity in MET-minutes/week by summing the walking, moderate, and vigorous MET-minutes/week scores.

### Statistical analysis

We used unpaired *t* and chi-square tests to assess whether there were differences between the groups of participants who lived alone and those living with someone. We conducted linear regression analyses of domestic electricity use. In our analysis of temperature sensitivity, we averaged the independent variables throughout the year, and we used those averages for linear regression analyses. Among the independent variables, we employed annual averages for systolic blood pressure, diastolic blood pressure, body mass index, body fat, and step count. With respect to the variables obtained from the questionnaire, we used average values of the valid responses. The independent variables employed in the regression model included age and gender for Table 2 and age, gender, and number of household inhabitants for Table 3. We undertook all statistical analysis using SPSS version 20.0 (International Business Machines Corporation, Armonk, New York, USA).

## Results

### Participant characteristics by family unit

**Figure 3:**
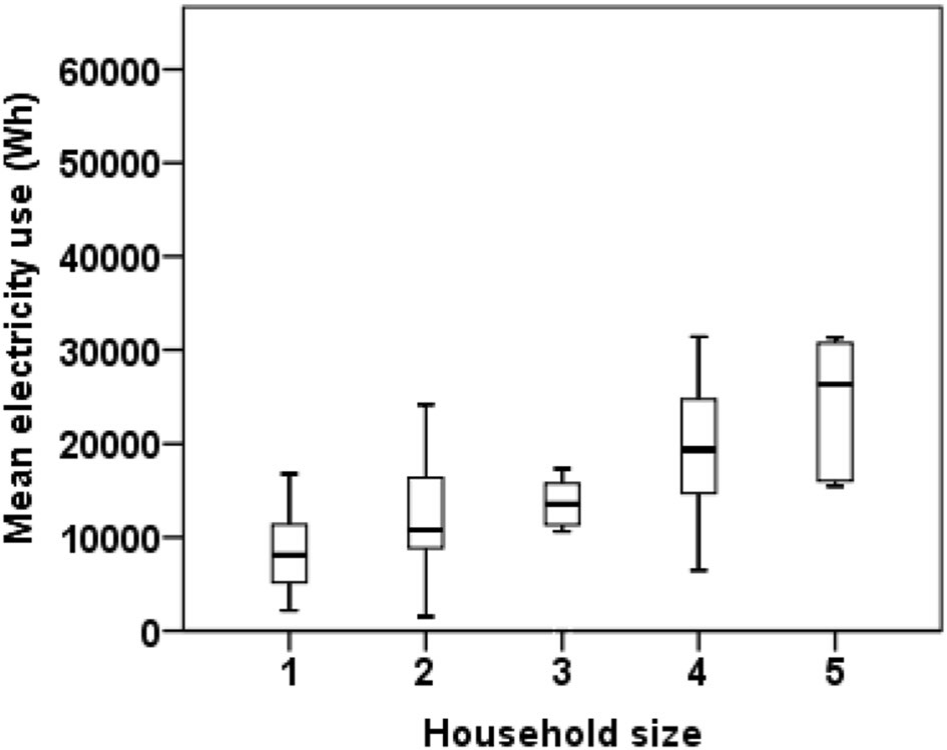
Mean electricity use by household size. The box plot shows the mean electricity use per day (Wh) among all participants classified by household size.

Table 1 shows the characteristics of the participants by family unit. Regarding participants living alone and those living with someone, we observed no significant differences in the following: mean age; years of education; smoking status; vegetable or fruit intake per week; alcohol consumption per month; total METs; minutes of inactivity per day; presences of diabetes or hypertension; waist circumference; and blood HDL, low-density lipoprotein (LDL) and neutral fat levels. Figure 3 shows the difference in mean electricity use by household size. As expected, the median electricity use increased with household size: 8113 Wh for living alone; 11,464 Wh for two occupants; 13,918 Wh for three occupants; 18,129 Wh for four occupants; and 26,329 Wh for five occupants.

**Table 1:** Participant characteristics by family unit.

	Living alone	Living with someone	P*
n (%)	41 (37.3)	64 (58.2)	
Mean age (standard deviation [SD])	39.3 (9.3)	40.7 (8.9)	0.436
Years of education (SD)	16.2 (2.8)	16.8 (2.2)	0.226
**Smoking status**			
Current smoker, n (%)	13 (31.7)	13 (20.3)	0.186
Ex-smoker, n (%)	5 (12.2)	16 (25.0)	
Never smoker, n (%)	23 (56.1)	35 (54.7)	
Vegetable intake/week, cups (SD)	8.5 (8.8)	11.2 (7.5)	0.132
Fruit intake/week, cups (SD)	3.2 (6.4)	3.2 (3.5)	0.981
Alcohol consumption/month, g (SD)	396.3 (452.3)	2674.5 (9338.6)	0.082
Total MET-minutes/week (SD)	980.4 (946.6)	1343.6 (1387.6)	0.149
Inactive minutes/day on weekdays (SD)	424.4 (317.2)	495.0 (273.0)	0.244
Inactive minutes/day at weekends (SD)	410.3 (257.0)	425.6 (249.0)	0.763
**Comorbidity**			
Diabetes presence, n (%)	5 (12.2)	2 (3.1)	0.069
Hypertension presence, n (%)	4 (10.0)	12 (18.8)	0.229
Waist circumference, cm (SD)	79.1 (12.0)	80.4 (8.2)	0.532
HDL, mg/dl (SD)	59.4 (16.0)	61.6 (15.0)	0.519
LDL, mg/dl (SD)	111.6 (24.9)	113.6 (33.5)	0.766
Neutral fat, mg/dl (SD)	110.3 (62.0)	119.6 (115.9)	0.669

*Indicates comparison between participants living alone and living with someone using *t* or chi-square tests.

### Analysis of domestic electricity use among participants living alone

Table 2 presents the significant variables by linear regression models for participants living alone. The mean electricity use among participants living alone from 20 February to 11 March 2013 was negatively associated with HDL level (*P* = 0.003) and positively associated with LDL (*P* = 0.003) and neutral fat levels (*P* = 0.001). The linear relationships remained significant (*P* = 0.008 for HDL, *P* = 0.007 for LDL, and *P* = 0.020 for neutral fat) after adjusting for age and gender.

**Table 2:** Electricity use and health status among participants living alone (n = 41).

	Unstandardized coefficients	95% confidence interval	*P* value	Unstandardized coefficients*	95% confidence interval	*P* value
	B			B		
HDL, mg/dl	− 165.95	− 270.60, − 61.29	0.003	− 156.69	− 268.04, − 45.34	0.008
LDL, mg/dl	114.82	44.48, 185.17	0.003	101.54	31.51, 171.57	0.007
Neutral fat, mg/dl	50.22	22.41, 78.02	0.001	42.18	7.49, 76.87	0.020

*Indicates adjusted unstandardized coefficient B from linear regression models including age and gender.

### Analysis of domestic electricity use among all participants

Table 3 displays the statistical results by linear regression models for all participants. The mean electricity use among all participants from 20 February to 11 March 2013 was positively associated with monthly alcohol intake (*P* = 0.033) and negatively associated with mean step count (*P* = 0.024). The linear relationships remained significant (*P* = 0.040 for mean step count, *P* = 0.028 for monthly alcohol intake) after adjusting for age, gender, and household size. After adjusting for confounding valuables, the mean electricity use among all participants was positively associated with the LDL/HDL ratio (*P* = 0.017) and negatively associated with vegetable intake per week (*P* = 0.044).

**Table 3:** Electricity use and health status among all participants (n = 105).

	Unstandardized coefficients	95% confidence interval	*P* value	Unstandardized coefficients*	95% confidence interval	*P* value
	B			B		
BMI	127.04	− 552.47, 806.55	0.372	187.76	− 452.52, 828.03	0.561
Body fat, %	139.73	− 214.44, 493.91	0.435	− 5.89	− 333.18, 321.41	0.972
Abdominal circumference, cm	61.82	− 150.89, 274.53	0.565	87.38	− 135.26, 310.03	0.437
Systolic blood pressure, mmHg	− 39.31	− 220.25, 141.63	0.667	9.36	− 169.32, 188.04	0.917
Diastolic blood pressure, mmHg	− 34.42	− 223.91, 155.06	0.719	− 71.08	− 254.62, 112.45	0.443
Heart rate	91.92	− 140.54, 324.39	0.434	178.22	− 26.07, 382.51	0.086
HDL, mg/dl	− 119.00	− 253.21, 15.22	0.081	− 129.78	− 262.37, 2.81	0.055
LDL, mg/dl	37.07	− 29.91, 104.05	0.274	39.98	− 23.16, 103.12	0.211
Neutral fat, mg/dl	6.87	− 2.78, 36.52	0.091	16.26	− 1.93, 34.44	0.079
LDL/HDL ratio	2443.73	− 16.01, 4903.47	0.051	2845.5	533.8, 5157.2	0.017
Mean step count	− 0.50	− 0.93, − 0.07	0.024	− 0.53	− 1.03, − 0.026	0.040
Total MET	− 0.62	− 2.20, 0.97	0.442	− 0.96	− 2.33, 0.41	0.167
Vegetable intake, cups	− 194.79	− 393.91, 4.32	0.055	− 205.18	− 405.13, − 5.24	0.044
Alcohol consumption, mg	0.005	0.000, 0.009	0.033	0.004	0.000, 0.008	0.028
Cigarettes smoked per day	87.49	− 310.47, 485.47	0.658	112.88	− 272.61, 498.37	0.555

*Indicates adjusted unstandardized coefficient B from linear regression models including age, gender, and household size.

### Analysis of temperature sensitivity in winter

Factors affecting temperature sensitivity in winter and summer appear in Tables 4 and 5, respectively. We defined temperature sensitivity in winter as the negative slope in Fig. 2b obtained from the linear fitting below 20 °C. Thus, a lower value for temperature sensitivity in winter signified greater domestic electricity use linearly with decreasing outside temperature. After adjusting for age and household size among men, temperature sensitivity in winter was negatively related to the LDL/HDL ratio (*P* = 0.022) and alcohol intake per month (*P* = 0.004). After adjusting for age and household size among women, temperature sensitivity in winter was negatively associated with the LDL/HDL ratio (*P* = 0.033) and vegetable intake per week (*P* = 0.034); after the same adjustment among women, temperature sensitivity in winter was positively related to body fat percentage (*P* = 0.024) and abdominal circumference (*P* = 0.031).

**Table 4:** Association between temperature sensitivity of electricity use and vital and lifestyle data in winter.

	Men			Women		
	Unstandardized coefficients	95% confidence interval	*P* value	Unstandardized coefficients	95% confidence interval	*P* value
	B			B		
Body fat, %	0.003	− 0.023, 0.029	0.812	0.038	0.005, 0.070	0.024
Abdominal circumference, cm	− 0.005	− 0.019, 0.010	0.498	0.025	0.003, 0.048	0.031
LDL/HDL ratio	− 0.086	− 0.159, − 0.013	0.022	− 0.076	− 0.146, − 0.006	0.033
Vegetable intake, cups	0.000	− 0.018, 0.019	0.971	− 0.027	− 0.051, − 0.002	0.034
Alcohol consumption, mg	− 0.029	− 0.049, − 0.010	0.004	0.040	− 0.045, 0.126	0.329

Adjusted unstandardized coefficient B from linear regression models including age and household size.

**Table 5:** Association between temperature sensitivity of electricity use and vital and lifestyle data in summer.

	Men			Women		
	Unstandardized coefficients	95% confidence interval	*P* value	Unstandardized coefficients	95% confidence interval	*P* value
	B			B		
Body fat, %	0.020	− 0.005, 0.045	0.107	− 0.051	− 0.086, − 0.016	0.007
BMI	0.039	0.002, 0.076	0.037	− 0.084	− 0.149, − 0.019	0.014
Abdominal circumference, cm	0.010	− 0.004, 0.024	0.158	− 0.030	− 0.056, 0.004	0.024
Vegetable intake/week, cup	0.033	0.010, 0.055	0.005	0.028	0.007, 0.048	0.008

Adjusted unstandardized coefficient B from linear regression models including age and household size.

### Analysis of temperature sensitivity in summer

We defined the positive slope in Fig. 2b obtained from the linear fitting over 25 °C as the temperature sensitivity in summer. Thus, a higher value for temperature sensitivity in summer signified greater domestic electricity use linearly with increasing outside temperature. After adjusting for age and household size among men, temperature sensitivity in summer was positively associated with body mass index (BMI; *P* = 0.037) and vegetable intake per week (*P* = 0.005). After the same adjustments among women, temperature sensitivity in summer was negatively associated with body fat percentage (*P* = 0.007), BMI (*P* = 0.014), and abdominal circumference (*P* = 0.024); it was positively associated with vegetable intake per week (*P* = 0.008).

## Discussion

Through a home health support system with wireless electricity monitoring, we verified in this study the relationship between domestic electricity use and vital data and lifestyle. Interestingly, mean electricity use at the end of winter was associated with vital data (such as blood lipid level) and lifestyle (such as vegetable and alcohol intake and step counts). Temperature sensitivity in winter and summer was related to vital data (such as the LDL/HDL ratio, BMI, body fat, and abdominal circumference) and lifestyle (such as vegetable and alcohol intake).

Mean electricity use per day among participants living alone was negatively associated with HDL level and positively associated with LDL and natural fat levels; among participants living with someone, it was positively associated with alcohol intake and the LDL/HDL ratio and negatively associated with vegetable intake and mean step count. Almost all the participants had a heating appliance in their homes. The daily minimum temperatures in the study period were under 13 °C, so participants would have used a heating appliance to increase the indoor temperature. Participants with a high intake of oily food and alcohol and low intake of vegetables and who were physically inactive outside may have led a sedentary life at home in rooms warmed with a heating appliance, thereby leading to high electricity use.

Temperature sensitivity in winter for men was negatively associated with the LDL/HDL ratio and alcohol consumption; for women, it was positively associated with body fat and abdominal circumference and negatively associated with the LDL/HDL ratio and vegetable intake. Male participants with a high intake of oily food and alcohol may have used heating appliances at higher power and for longer to keep their living space warm and comfortable with decreasing outside temperature. Slender female participants may have used heating appliances at higher power to warm themselves with decreasing outside temperature. Temperature sensitivity in summer among men was positively associated with BMI and vegetable intake; among women, it was negatively associated with body fat, BMI, and abdominal circumference, and it was positively associated with vegetable intake. Consistent with the association of temperature sensitivity in winter, slender females may have used air conditioners at higher power to keep comfortable with increasing outside temperature. Both men and women with high vegetable intake would probably be health-conscious individuals. Such people would probably also be careful not to get heat stroke and keep their living space cool to avoid heat stress.

This study determined that daily domestic electricity use may be employed as an index for evaluating health with respect to lifestyle-related diseases. Domestic electricity use is reportedly associated with household income^17,18^. In this study, participants were not required to indicate household income in the questionnaire. However, we assume that the financial level of the participants was comparatively high because having an Internet environment at home was required to participate in this study; almost all the participants had an air conditioner and heating appliance. In addition to household income, residential environment (wooden or reinforced concrete structure, living floor space, and direction of window or balcony) and power-saving consciousness could be factors worth considering. Domestic electricity use may also be influenced by household style (such as number and age of children or seniors); however, household style was not considered in this study owing to the small sample size. In regions with a warmer or cooler climate, temperature sensitivity and domestic electricity use in winter or summer may differ from our findings. Differences in the relationship between domestic electricity use and vital data and lifestyle according to household style and region should be investigated in future.

One issue of selection bias should be acknowledged because our sampling method was not random. The study participants had comparatively high financial levels and were health-conscious individuals: that could have an impact on the generalizability of the results. However, this research was not a comparative study; thus, the selection process itself should not have had a direct impact on the findings. To validate our results, we intend to undertake a future study using a larger sample collected at random.

In the present study, we determined that home electricity use was closely associated with important factors in preventing lifestyle-related diseases. Such diseases are the main cause of death in the world; thus, it is essential to check lifestyles regularly and improve them as appropriate. Prolonged time spent at home owing to the novel coronavirus pandemic could elevate the risk for lifestyle-related diseases. Accordingly, we believe that the finding of home electricity use being closely associated with important factors for preventing lifestyle-related diseases constitutes beneficial information from a public health perspective. Regarding real-world application, recording vital data (such as blood pressure, body weight, and blood lipid levels) and lifestyle-related data (such as vegetable intake, alcohol consumption, number of cigarettes smoked, and step count) is time consuming; by contrast, recording electricity use presents no burden because the information is collected by an electric power company. Further, daily electricity use can be indicated to residents by IT visualization. If the health risk assessed through residents’ electricity use could be shared with regional medical institutions collaborating with the power company, it would help enhance the general health level of the region. To achieve that goal, the relationship between home electricity use and risk of lifestyle-related diseases deserves future elucidation.

In conclusion, this study determined for the first time the relationship between daily domestic electricity use and vital data (blood lipid levels) and lifestyles (alcohol or vegetable intake and step counts) using a home health support system with wireless electricity monitoring. Temperature sensitivity and electricity use in winter and summer were related to vital data (LDL/HDL ratio, body fat percentage, BMI, and abdominal circumference) and lifestyle (alcohol or vegetable intake).
